# Quality of life comparison between esophagogastrostomy and double tract reconstruction for proximal gastrectomy assessed by Postgastrectomy Syndrome Assessment Scale (PGSAS)‐45

**DOI:** 10.1002/ags3.12645

**Published:** 2022-12-15

**Authors:** Masami Ikeda, Nobuhiro Takiguchi, Takayuki Morita, Hisahiro Matsubara, Atsushi Takeno, Akinori Takagane, Kazutaka Obama, Atsushi Oshio, Koji Nakada

**Affiliations:** ^1^ Department of Surgery Asama General Hospital Saku‐shi Japan; ^2^ Division of Gastrointestinal Surgery Chiba Cancer Center Chiba Japan; ^3^ Department of Surgery, Hokkaido Gastroenterology Hospital Sapporo‐shi Japan; ^4^ Department of Frontier Surgery, Graduate School of Medicine Chiba University Chiba Japan; ^5^ Department of Surgery Osaka National Hospital Osaka Japan; ^6^ Department of Surgery Hakodate Goryoukaku Hospital Hakodate‐shi Japan; ^7^ Department of Surgery, Graduate School of Medicine Kyoto University Kyoto Japan; ^8^ Faculty of Letters, Arts and Sciences Waseda University Tokyo Japan; ^9^ Department of Laboratory Medicine The Jikei University School of Medicine Tokyo Japan

**Keywords:** double tract, esophagogastrostomy, gastric cancer, proximal gastrectomy, quality of life

## Abstract

**Aim:**

The current study compared the postoperative quality of life (QOL) between the esophagogastrostomy method (PGEG) and double tract method (PGDT) after proximal gastrectomy using the Postgastretomy Syndrome Assessment Scale (PGSAS)‐45.

**Methods:**

Among the 2364 patients who received the PGSAS‐45 questionnaire, 300 PGEG and 172 PGDT cases responded. The main outcomes measures (MOMs) consisted of seven subscales (SS) covering symptoms, meals (amount and quality), ability to work, dissatisfaction with daily life, physical and mental component summary of the 8‐Item Short Form Health Survey (SF‐8), and change in body weight, and were compared between PGEG and PGDT.

**Results:**

Overall, PGDT promoted significantly better constipation SS scores (*p* < 0.05), whereas PGEG tended to promote better body weight (BW) loss% (*p <* 0.10). A stratified analysis based on the remnant stomach size revealed that among those with a remnant stomach size of 1/2, PGDT had significantly better constipation and dumping SS scores (*p* < 0.05) and tended to have better working conditions (*p* < 0.10) compared to PGEG. Even among those with the remnant stomach size of 2/3, PGDT had significantly better diarrhea SS scores, lesser dissatisfaction with symptoms, and better dissatisfaction with daily life SS scores (*p* < 0.05) and tended to have better constipation SS scores and lesser dissatisfaction with work (*p <* 0.10) compared to PGEG.

**Conclusions:**

After comparing the QOLs of PGEG and PGDT, the stratified analysis according to remnant stomach sizes of 1/2 and 2/3 revealed that PGDT was relatively superior to PGEG for several MOMs.

## Graphical abstract

The stratified analysis according to remnant stomach sizes of 1/2 and 2/3 after proximal gastrectomy revealed that the double tract method was relatively superior to the esophagogastrostomy for QOL.

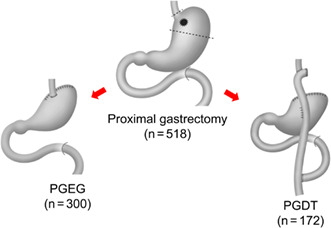

## 1 INTRODUCTION

Advances in diagnostic and therapeutic techniques for gastric cancer have improved the early diagnosis of gastric cancer patients and prognosis of advanced gastric cancer.^1^, ^2^, ^3^, ^4^ As such, the long‐term quality of life (QOL) after gastrectomy has also required attention. Unfortunately, both Japan and Western countries have seen an increase in the incidence of cancers in the upper third of the stomach and gastroesophageal junction.^5^, ^6^, ^7^ In Japan, the incidence of gastric cancer has decreased due to the decrease in the rate of *H. pylori* infection and the spread of *H. pylori* infection detection/eradication treatment in health checkups.^8^ In such situations there is an increase in the rate of the upper gastric cancer.^9^ Proximal gastrectomy has been a frequent treatment approach for early upper third gastric cancer. Various reconstruction methods have been used for proximal gastrectomy. Among them, the esophagogastrostomy method (PGEG) and double tract method (PGDT) have recently been the most commonly utilized reconstruction methods in Japan. However, it remains unclear which reconstruction method is better for postoperative QOL.

A large retrospective study using the Postgastrectomy Syndrome Assessment Scale (PGSAS‐45) reported that proximal gastrectomy promoted lower postgastrectomy burden than total gastrectomy with Roux‐en‐Y reconstruction.^10^ Hence, proximal gastrectomy has become a potential option as a function‐preserving surgery for early upper third gastric cancer.^11^ Various reconstruction methods after proximal gastrectomy have been proposed for preventing esophageal reflux and achieving optimal food storage and outflow from the remnant stomach, which are considered crucial for maintaining postoperative QOL after proximal gastrectomy. A nationwide multi‐institutional surveillance study (“PGSAS NEXT” study) was conducted to investigate the optimal gastrectomy procedures for improving postoperative QOL in patients with the cancer located in the upper third of the stomach or around the esophagogastric junction. Based on collected data, we compared the postoperative QOLs after proximal gastrectomy for patient with gastric cancer located in the upper third of the stomach according to the reconstruction procedures, esophagogastrostomy method (PGEG), and double tract method (PGDT).

## 2 METHODS

### 2.1 Patients

The patient inclusion criteria were as follows: (1) females or males aged 20 years or older; (2) cancer located at the upper third of the stomach or around the esophagogastric junction regardless of stage or histologic type; (3) achieved R0 resection; (4) no recurrence or metastasis; (5) more than 6 months after gastrectomy; (6) more than 6 months after the termination of former chemotherapy; (7) underwent gastrectomy only once; (8) performance status 0 or 1; (9) capable of understanding the questionnaire; (10) no other disease or previous surgery that may influence the results of the questionnaire aside from gastrectomy; (11) no organ failure or mental disease; (12) spontaneous agreement of the said person.

The patient exclusion criteria were as follows: (1) active dual malignancy; (2) synchronous surgery with exception of the resection or extraction of the perigastric organs to accomplish gastrectomy or lymph node dissection, as well as that equivalent to cholecystectomy.

### 2.2 Data of case report forms

Patient and surgical information [age, sex, height, BW, postoperative period, approach (laparotomy/laparoscope), celiac branch of vagal nerve preservation (yes/no), chemotherapy history, clinical stage, extent of lymph node dissection, information on simultaneous organ resection, details of surgical procedures, etc.] was collected from case report forms.

### 2.3 QOL assessment using the PGSAS‐45 questionnaire

**FIGURE 1:**
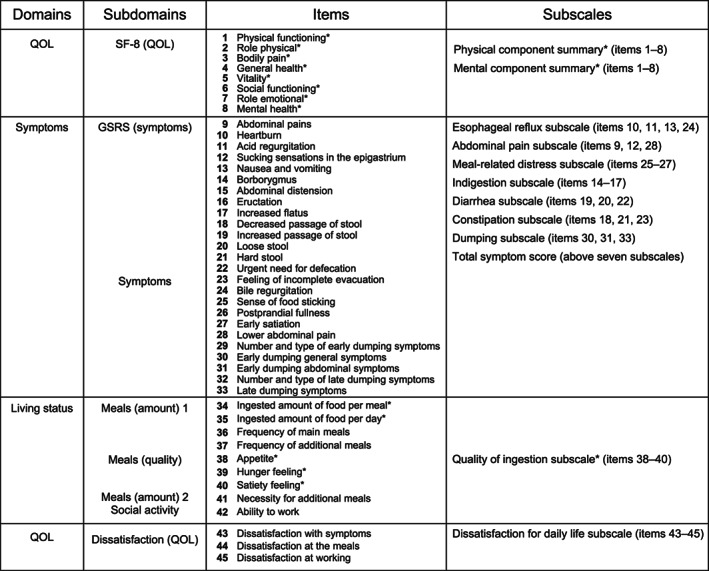
Structure of Postgastrectomy Syndrome Assessment Scale (PGSAS)‐45 (domains/subdomains/items/subscales). In items or subscales with*; higher score indicating better condition. In items or subscales without*; higher score indicating worse

The PGSAS‐45 is composed of questions pertaining to 45 items (Figure 1), which include eight items from the existing the 8‐Item Short Form Health Survey (SF‐8),^12^ 15 items from the Gastrointestinal Symptom Rating Scale (GSRS),^13^, ^14^ and 22 items determined to be clinically important and newly selected by surgeons affiliated with the Japanese Postgastrectomy Syndrome Working Party. Factor analysis was performed in our previous Postgastrectomy Syndrome Assessment Study (PGSAS study), and 23 symptom items were clustered into the following seven subscales (SSs): esophageal reflux, abdominal pain, meal‐related distress, indigestion, diarrhea, constipation, and dumping.^15^ Moreover, the total symptom score; quantity of each meal; necessity for additional meals; quality of ingestion SS; working conditions; dissatisfaction with symptoms, meals, and work; dissatisfaction with daily life SS; the physical component summary (PCS) and mental component summary (MCS) of the SF‐8; and change in BW were determined as the main outcome measures. The SS scores represented the average scores for the component items or SSs except PCS and MCS of the SF‐8. For items 1–8, 34, 35, and 38–40, higher scores indicated better outcomes, whereas for items 9–28, 30, 31, 33, and 41–45, higher scores indicated worse outcomes.

### 2.4 Study methods

This study utilized continuous sampling from a central registration system for participant enrollment. The questionnaire was distributed to eligible patients. Patients were instructed to return completed forms to the data center. All QOL data from questionnaires were matched with individual patient data collected via case report forms.

The methods used for measuring the distance from the diaphragm to the esophago‐gastrointestinal anastomosis were defined as follows. The anastomotic site was confirmed by the staple line on the axial cross‐sectional computed tomography (CT) image. The midpoint between the top slice where the esophageal hiatus of the diaphragm can be depicted and the bottom slice where it cannot be confirmed on the CT image was defined as the diaphragm level (D, 0 mm). The distance between the diaphragm and esophago‐gastrointestinal anastomosis was assigned a positive or negative value depending on whether the latter was located below or above the diaphragm, respectively.

This study was registered with the University Hospital Medical Information Network's Clinical Trials Registry (UMIN‐CTR; registration number 000032221) and was approved by the ethics committees of all institutions.

### 2.5 Statistics

Patient characteristics and main outcome measures were compared between PGEG and PGDT using *t*‐tests and Fisher's exact tests. All outcome measures were further analyzed using multiple regression analyses. Ten factors—reconstruction method, age, sex, postoperative period, operative approach, preservation of the celiac branch of the vagus, chemotherapy, clinical stage, extent of lymph node dissection, combined resection—were included in the multiple regression analysis as explanatory variables. These factors were selected according to their clinical importance and based on the results of previous Postgastrectomy Syndrome Assessment Studies. Statistical significance was set at *p* < 0.05. Where multiple regression analysis yielded a *p*‐value <0.1, the standardization coefficient of regression (*β*) and the *p*‐value are shown in a table. Cohen's *d*, *β*, and *R* ^*2*^ were used to measure the effect sizes. Interpretation of effect sizes were ≥0.2 = small, ≥0.5 = medium, and ≥0.8 = large in Cohen's *d*; ≥0.1 = small, ≥0.3 = medium, and ≥0.5 = large in *β*; and ≥0.02 = small, ≥0.13 = medium, and ≥0.26 = large in *R* ^*2*^. All statistical analyses were performed using JMP12.0.1 software (SAS Institute Inc.).

## 3 RESULTS

### 3.1 Retrieving the questionnaire

**FIGURE 2:**
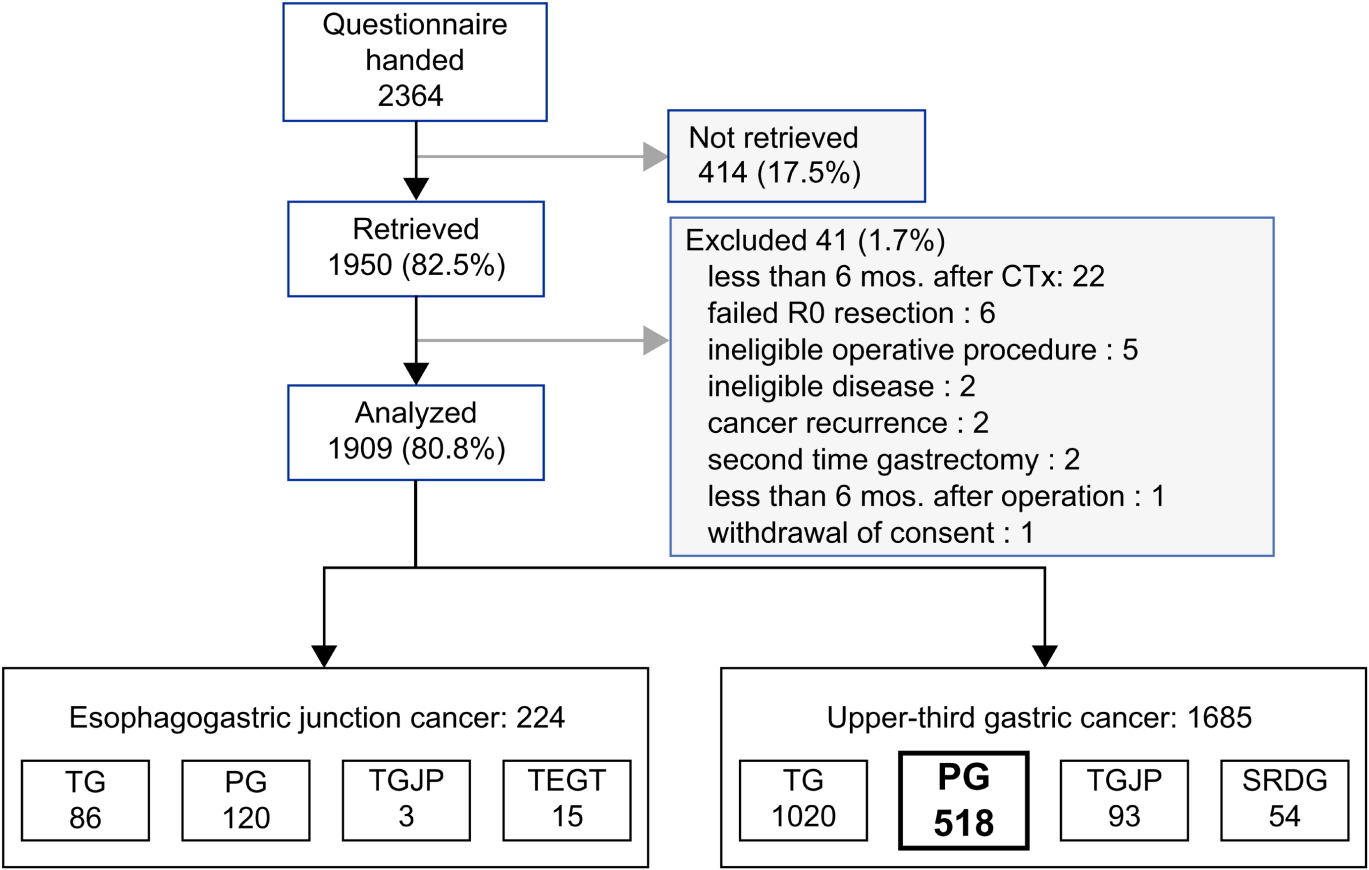
Outline of the study. CTx, chemotherapy; PG, proximal gastrectomy; SRDG, small remnant distal gastrectomy; TEGT, thoracic esophagectomy with gastric‐tube reconstruction; TG, Total gastrectomy; TGJP, Total gastrectomy with jejunal pouch reconstruction

Between July 2018 and December 2019, the PGSAS‐45 questionnaire was distributed to 2364 patients across the 70 institutions that participated in this study. Among those who received a questionnaire, 1950 (82.5%) responded, of which 41 were deemed ineligible. As a result, 1909 questionnaires (80.8%) were ultimately included for analyses related to the PGSAS NEXT study. Among the 1909 patients included, 518 had undergone proximal gastrectomy for upper‐third gastric cancer (Figure 2).

**FIGURE 3:**
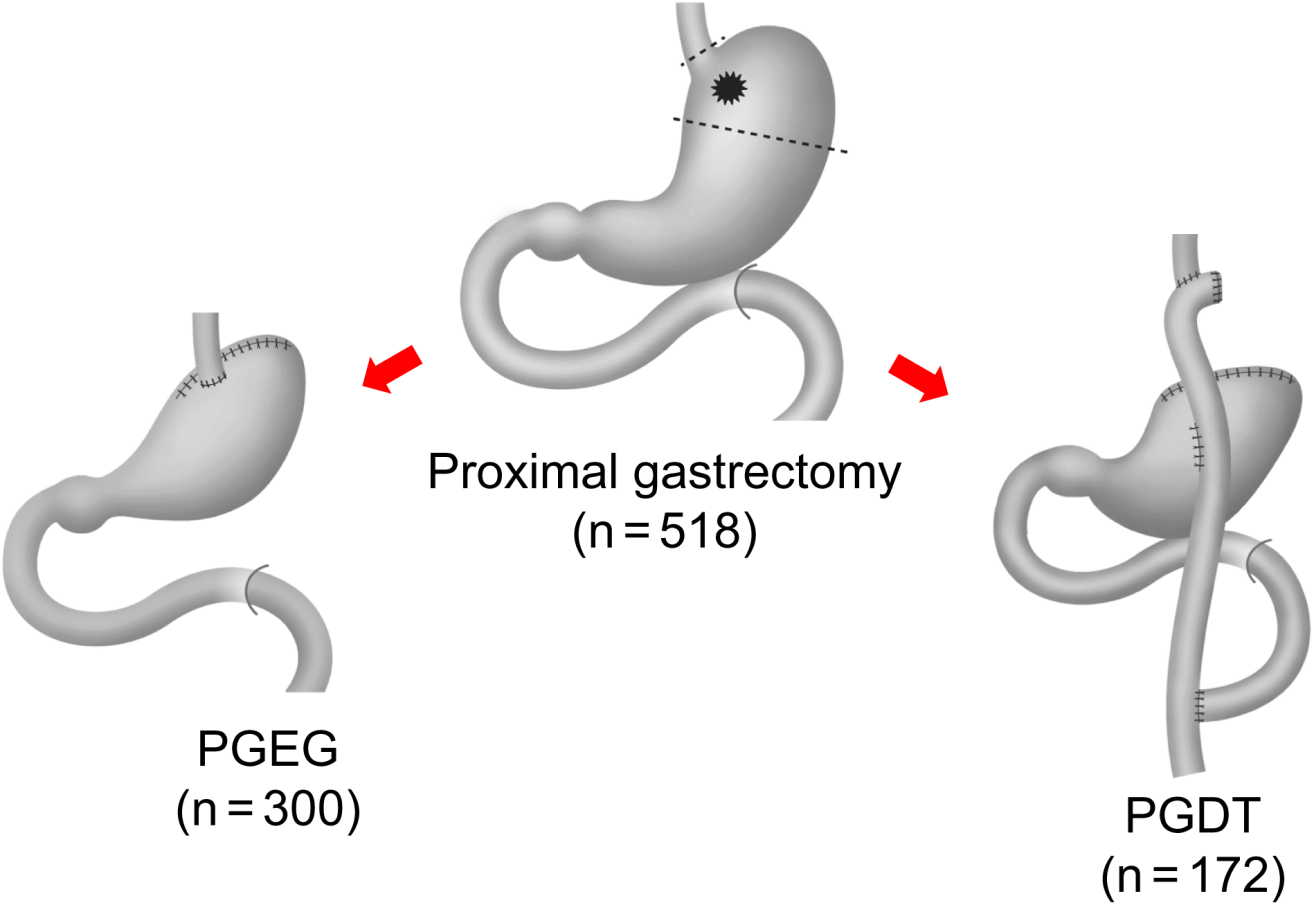
Schema of esophagogastrostomy (PGEG) and double tract (PGDT) reconstructions after proximal gastrectomy (Medical Illustration by Hiromitsu Yokota)

The current study analyzed 300 patients and 172 who had undergone PGEG and PGDT, respectively (Figure 3). Other reconstruction methods used for proximal gastrectomy included only 30 cases of jejunal interposition and 16 cases of jejunal pouch interposition and were considered insufficient for analysis.

The PGEG group was consisted of 276 cases who received the anti‐reflux method (double flap method, 153 cases; creation of pseudofornix and/or Hisoid angle, 67 cases; fundoplication method, 44 cases; side overlap with fundoplication using the Yamashita [SOFY] method,^16^ 11 cases; other procedures, 11 cases), 21 cases who did not receive the anti‐reflux method, and three unknown cases. Reconstruction procedures were not regulated by the protocol and depended on the principle of the institution or discretion of each surgeon.

### 3.2 Patient characteristics

Patient characteristic of both groups are summarized (Table 1). The PGDT group was younger than the PGEG group (PGEG, 70.8 years old; PGDT, 68.6 years old; *p* = 0.013). The esophageal resection length was longer in the PGDT group (PGEG, 4.1 ± 6.5 mm; PGDT, 7.2 ± 8.2 mm; *p <* 0.001), whereas the distance between the anastomosis site and the diaphragm was shorter in the PDGT group (PGEG, 7.2 ± 16.0 mm; PGDT, 1.0 ± 14.4 mm; *p* < 0.001).

**TABLE 1:** Patients' characteristics

	PGEG (*n* = 300) mean (SD)	PGDT (*n* = 172) mean (SD)	*t*‐test *p ‐*value
Age (year)	70.8 (9.5)	68.6 (9.3)	0.013
Preoperative BMI (kg/m^2^)	22.8 (3.0)	23.0 (3.2)	0.446
Postoperative BMI (kg/m^2^)	20.8 (2.8)	19.9 (2.5)	0.287
Postoperative period (month)	40.3 (33.8)	39.3 (22.1)	0.720
Length of esophageal resection (mm)	4.1 (6.5)	7.2 (8.2)	<0.001
Distance from diaphragm to anastomosis (mm)	7.2 (16.0)	1.0 (14.4)	<0.001

	PGEG (*n* = 300)	PGDT (*n* = 172)	Fisher's exact test *p ‐*value		PGEG (*n* = 300)	PGDT (*n* = 172)	Fisher's exact test *p ‐*value
Gender			0.232	cStage (JGCA 14th)			0.745
Male	237	128		I	282	163	
Female	63	44		IIA/IIB	9	7	
Abdominal approach			0.001	III	7	2	
Open	96	31		IVA/IVB	1	0	
Laparoscopy	204	141		Chemotherapy			0.529
Celiac branch of vagus			<0.001	Postoperative	20	9	
Preserved	77	15		Pre and postoperative	2	0	
Divided	216	152		None	278	163	
Tumor location (JGCA 14th)			0.026	Extent of lymphnode dissection			0.259
UE (Siewert type III)	6	4		D0	3	0	
U	284	152		D1	25	14	
UM	8	9		D1+	265	149	
MU	2	7		D2	6	8	
Extent of esophageal resection			0.001	Combine resection			0.548
Lower thoracic	5	1		None	278	157	
Abdominal	144	112		Gallbladder	19	15	
None	149	59		Spleen	2	0	
Level of esophago‐GI anastomosis			0.002	Pancreas	1	0	
Ti	39	23		Others	0	0	
D	106	89		Size of remnant stomach			<0.001
A	148	59		Greater than or equal to 3/4	57	1	
				Around 2/3	165	60	
				Around 1/2	73	97	
				Around 1/3	0	13	

Abbreviations: BMI, body mass index; PGEG, Proximal gastrectomy with esophagogastrostomy reconstruction; PGDT, Proximal gastrectomy with double tract reconstruction; SD, standard deviation.

Laparoscopic surgery was common in both groups, whereas the rate of laparoscopic surgery was higher in the PGDT group than in the PGEG group (PGEG, 68%; PGDT, 82.0%; *p* = 0.001). The preservation rate of the celiac branch of vagus nerve was lower in the PGDT group than in the PGEG group (PGEG, 27.2%; PGDT, 9.0%; *p <* 0.001). Proximal gastrectomy was mainly localized in the U region and cStage I. D1 + or less lymph node dissection was selected in almost all cases. Combined resection was performed in 22 cases (7.3%) in the PGEG group and 15 cases (8.7%) in the PGDT group and mainly involved the gall bladder.

The size of the remnant stomach was significantly associated with the selection of reconstruction method (i.e., PGEG or PGDT). The size of the remnant stomach was significantly smaller in the PGDT group. Cases with a remnant stomach of ≥3/4 accounted for 57 cases (98.3%) in the PGEG group and one case (1.7%) in the PGDT group. Cases with a remnant stomach of 2/3 accounted for 165 cases (73.3%) in the PGEG group and 60 cases (26.7%) in the PGDT group. Cases with a remnant stomach of 1/2 accounted for 73 cases (42.9%) in the PGEG group and 97 cases (57.1%) in the PGDT group. In contrast, only 13 cases had a remnant stomach of 1/3, all of whom were in the PGDT group.

### 3.3 Comparison of postoperative QOL between PGEG and PGDT

The main outcome measures of PGSAS‐45 were compared between the PGEG and PGDT groups (Table 2). Notably, the PGDT group tended to have had greater BW loss than the PGEG group (PGEG, 11.4% vs. PGDT, 13.0%; *p* = 0.052; Cohen's *d* = 0.19). However, the constipation SS was significantly lower in the PGDT group than in the PGEG group (PGEG, 2.5 vs. PGDT, 2.1; *p* = 0.002; Cohen's *d* = 0.30), which indicated better status in the PGDT group. No significant differences in the other main outcome measures were observed. Multiple regression analysis was also performed to adjust the effects of multiple clinical factors including approaches, but the effect of the reconstruction method on the postoperative QOL for proximal gastrectomy was almost the same as the result of univariate analysis (Table 3).

**TABLE 2:** Comparison of postoperative QOL between PGEG and PGDT (all cases of PGEG and PGDT)

Domain	Main outcome measures	PGEG (*n* = 300)		PGDT (*n* = 172)		*t*‐test	Cohen's *d*
		Mean	SD	Mean	SD	*p‐*value	
Symptoms	Esophageal reflux SS	1.9	1.0	2.1	1.0	0.156	
	Abdominal pain SS	1.7	0.8	1.8	0.8	0.658	
	Meal‐related distress SS	2.6	1.1	2.7	1.0	0.357	
	Indigestion SS	2.1	0.9	2.2	1.0	0.338	
	Diarrhea SS	2.3	1.3	2.2	1.2	0.572	
	Constipation SS	2.5	1.3	2.1	1.0	0.002	0.30
	Dumping SS	2.2	1.3	2.1	1.0	0.407	
	Total symptom score	2.2	0.9	2.2	0.7	0.844	
Living status	Change in BW*	−11.4%	8.4%	−13.0%	7.6%	0.052	0.19
	Ingested amount of food per meal*	6.2	1.8	6.3	1.9	0.767	
	Necessity for additional meals	2.2	0.9	2.3	0.9	0.598	
	Quality of ingestion SS*	3.6	1.0	3.7	1.0	0.725	
	Ability for working	2.1	1.0	2.1	1.0	0.929	
QOL	Dissatisfaction with symptoms	2.0	1.0	2.0	1.0	0.709	
	Dissatisfaction at the meal	2.6	1.2	2.7	1.1	0.400	
	Dissatisfaction at working	1.9	1.0	2.0	1.0	0.424	
	Dissatisfaction for daily life SS	2.2	0.9	2.2	0.9	0.613	
	PCS of SF‐8*	48.9	6.4	49.4	5.8	0.380	
	MCS of SF‐8*	49.6	6.0	49.5	5.8	0.820	

*Note*: Outcome measures with “*”: higher score indicating better condition. Outcome measures without “*”: higher score indicating worse condition. The interpretation of effect size in Cohen's *d*: ≥0.2 as small, ≥0.5 as medium, ≥0.8 as large.

Abbreviations: BW, body weight; MCS, mental component summary; PCS, physical component summary; SS, subscale; SD, standard deviation; PGEG, Proximal gastrectomy with esophagogastrostomy reconstruction; PGDT, Proximal gastrectomy with double tract reconstruction.

**TABLE 3:** Multiple regression analysis that coordinates the effects of multiple clinical factors

UGca		Reconstruction method [PGDT]		Age (Yrs)		Gender [male]		Postoperative period (Mons)		Approach [laparoscopic]		Celiac branch of vagus [preserved]		CTx [Y]		cStage [III/IV]		LN dissection [D1+]		LN dissection [D2/D2+]		Combined resection [Y]			
Domain	Main outcome measures	*β*	*p*‐value	*β*	*p*‐value	*β*	*p*‐value	*β*	*p*‐value	*β*	*p*‐value	*β*	*p*‐value	*β*	*p*‐value	*β*	*p*‐value	*β*	*p*‐value	*β*	*p*‐value	*β*	*p*‐value	*R* ^*2*^	*p*‐value
Symptoms	Esophageal reflux SS																							0.024	0.445
	Abdominal pain SS			−0.135	0.005											−0.085	0.090	−0.125	0.010					0.048	0.026
	Meal‐related distress SS			−0.154	0.001							−0.083	0.088					−0.082	0.086	−0.085	0.078			0.058	0.005
	Indigestion SS			−0.083	0.086			−0.097	0.052															0.039	0.089
	Diarrhea SS			−0.168	0.000													−0.087	0.065	−0.087	0.069	0.099	0.031	0.075	0.000
	Constipation SS	−0.153	0.002	0.105	0.029																			0.046	0.034
	Dumping SS			−0.147	0.003					0.106	0.052							−0.132	0.008					0.071	0.001
	Total symptom score			−0.104	0.036													−0.104	0.038					0.042	0.088
Living status	Change in BW*	−0.090	0.073			−0.114	0.018																	0.025	0.434
	Ingested amount of food per meal*											0.132	0.008											0.024	0.459
	Necessity for additional meals											−0.136	0.006											0.044	0.045
	Quality of ingestion SS*													0.081	0.098									0.032	0.219
	Ability for working			0.270	<0.0001																			0.083	<0.0001
QOL	Dissatisfaction with symptoms			−0.091	0.058	−0.108	0.021							0.104	0.031			−0.099	0.040					0.055	0.009
	Dissatisfaction at the meal			−0.103	0.031					0.143	0.007													0.057	0.006
	Dissatisfaction at working									0.094	0.084													0.023	0.488
	Dissatisfaction for daily life SS									0.126	0.019							−0.080	0.096					0.046	0.033
	PCS of SF‐8*			−0.193	<0.0001													−0.086	0.070	0.139	0.004			0.070	0.001
	MCS of SF‐8*															0.092	0.070							0.020	0.638

*Note*: Outcome measures with “*”: higher score indicating better condition. Outcome measures without “*”: higher score indicating worse condition. If *β* is positive, the score of the outcome measure of the patients belonging to the category in [brackets] is higher in cases when the factor is a nominal scale, and the score of outcome measure of the patients with larger values is higher in cases when the factor is a numeral scale.

Abbreviations: CTx, chemotherapy, LN, lymphnode, [Y], yes. **Table:** 

The interpretation of effect size	*β*	*R* ^*2*^
Small	≧0.1	≧0.02
Medium	≧0.3	≧0.13
Large	≧0.5	≧0.26

Subsequently, a stratified analysis based on the remnant stomach size was performed for those with a remnant stomach size of approximately 1/2 (Table 4) and 2/3 (Table 5), which were the main targets of proximal gastrectomy.

**TABLE 4:** Comparison of postoperative QOL between PGEG and PGDT (size of remnant stomach around 1/2)

Domain	Main outcome measures	PGEG (*n* = 73)		PGDT (*n* = 97)		*t*‐test	Cohen's *d*
		Mean	SD	Mean	SD	*p‐*value	
Symptoms	Esophageal reflux SS	2.1	1.1	2.0	0.9	0.843	
	Abdominal pain SS	1.9	0.9	1.7	0.8	0.106	
	Meal‐related distress SS	2.8	1.2	2.7	1.1	0.466	
	Indigestion SS	2.3	1.0	2.2	1.0	0.342	
	Diarrhea SS	2.4	1.5	2.3	1.3	0.650	
	Constipation SS	2.5	1.3	2.1	1.0	0.007	0.42
	Dumping SS	2.3	1.1	2.0	0.9	0.025	0.37
	Total symptom score	2.4	0.9	2.2	0.7	0.126	
Living status	Change in BW*	−12.4%	8.2%	−14.0%	8.1%	0.217	
	Ingested amount of food per meal*	6.0	1.8	6.2	1.9	0.478	
	Necessity for additional meals	2.4	1.0	2.3	0.9	0.460	
	Quality of ingestion SS*	3.5	1.1	3.7	0.9	0.163	
	Ability for working	2.3	1.1	2.0	1.0	0.098	0.26
QOL	Dissatisfaction with symptoms	2.2	1.1	2.0	1.0	0.243	
	Dissatisfaction at the meal	2.7	1.1	2.7	1.2	0.924	
	Dissatisfaction at working	2.0	1.1	2.0	1.0	0.899	
	Dissatisfaction for daily life SS	2.3	0.9	2.2	0.9	0.642	
	PCS of SF‐8*	48.0	6.1	49.5	5.9	0.111	
	MCS of SF‐8*	48.6	6.6	49.5	5.9	0.386	

*Note*: Outcome measures with “*”: higher score indicating better condition. Outcome measures without “*”: higher score indicating worse condition. The interpretation of effect size in Cohen's *d*: ≥0.2 as small, ≥0.5 as medium, ≥0.8 as large.

Abbreviations: BW, body weight; MCS, mental component summary; PCS, physical component summary; SS, subscale; SD, standard deviation; PGEG, Proximal gastrectomy with esophagogastrostomy reconstruction; PGDT, Proximal gastrectomy with double tract reconstruction.

**TABLE 5:** Comparison of postoperative QOL between PGEG and PGDT (size of remnant stomach around 2/3)

Domain	Main outcome measures	PGEG (*n* = 165)		PGDT (*n* = 60)		*t*‐test	Cohen's *d*
		Mean	SD	Mean	SD	*p‐*value	
Symptoms	Esophageal reflux SS	1.9	1.0	2.0	1.0	0.754	
	Abdominal pain SS	1.7	0.8	1.8	0.8	0.425	
	Meal‐related distress SS	2.6	1.1	2.6	1.0	0.668	
	Indigestion SS	2.1	0.9	2.2	1.0	0.623	
	Diarrhea SS	2.3	1.2	2.0	0.8	0.033	0.33
	Constipation SS	2.5	1.2	2.2	1.1	0.075	0.27
	Dumping SS	2.2	1.3	2.1	1.0	0.489	
	Total symptom score	2.2	0.8	2.1	0.7	0.437	
Living status	Change in BW*	−11.2%	8.1%	−11.2%	6.4%	0.982	
	Ingested amount of food per meal*	6.2	1.8	6.6	1.8	0.105	
	Necessity for additional meals	2.2	0.8	2.2	0.8	0.745	
	Quality of ingestion SS*	3.6	0.9	3.5	1.0	0.571	
	Ability for working	2.0	0.9	2.1	0.9	0.530	
QOL	Dissatisfaction with symptoms	2.1	1.0	1.8	0.9	0.050	0.30
	Dissatisfaction at the meal	2.7	1.2	2.4	1.0	0.111	
	Dissatisfaction at working	1.9	1.0	1.7	0.8	0.087	0.26
	Dissatisfaction for daily life SS	2.2	0.9	2.0	0.7	0.040	0.32
	PCS of SF‐8*	49.5	5.8	50.1	5.6	0.478	
	MCS of SF‐8*	49.8	5.3	49.7	5.5	0.914	

*Note*: Outcome measures with “*”: higher score indicating better condition. Outcome measures without “*”: higher score indicating worse condition. The interpretation of effect size in Cohen's *d*: ≥0.2 as small, ≥0.5 as medium, ≥0.8 as large.

Abbreviations: BW, body weight; MCS, mental component summary; PCS, physical component summary; PGEG, Proximal gastrectomy with esophagogastrostomy reconstruction; PGDT, Proximal gastrectomy with double tract reconstruction; SS, subscale; SD, standard deviation.

Among those with a remnant stomach size of around 1/2, the constipation SS (PGEG, 2.5 vs. PGDT, 2.1; *p* = 0.007; Cohen's *d* = 0.42) and dumping SS (PGEG 2.3 vs. PGDT 2.0; *p* = 0.025; Cohen's *d* = 0.37) were significantly lower in the PGDT group than that in the PGEG group, which indicated a better status in the PGDT group. Working ability tended to be lower in the PGDT group than in the PGEG group (PGEG, 2.4 vs. PGDT, 2.0; *p* = 0.098; Cohen's *d* = 0.26). No differences in the other main outcome measures, including body weight (BW) loss (%), were found between the two groups.

Among those with a remnant stomach size of around 2/3, the diarrhea SS (PGEG, 2.5 vs. PGDT, 2.0; *p* = 0.033; Cohen's *d* = 0.33), dissatisfaction with symptoms (PGEG, 2.1 vs. PGDT, 1.8; *p* = 0.050; Cohen's *d* = 0.30), and dissatisfaction with daily life SS (PGEG, 2.2 vs. PGDT, 2.0; *p* = 0.040; Cohen's *d* = 0.32) were significantly lower in the PGDT group than that in the PGEG group, which indicated a better status in the PGDT group. Additionally, the constipation SS (PGEG, 2.5 vs. PGDT, 2.2; *p* = 0.075; Cohen's *d* = 0.27) and dissatisfaction with work (PGEG, 1.9 vs. PGDT, 1.7; *p* = 0.087; Cohen's *d* = 0.26) tended to be lower in the PGDT group than in the PGEG group. No differences in the other main outcome measures, including BW loss (%), were found between the two groups.

## 4 DISCUSSION

The Japan Postgastrectomy Syndrome Working Party has created a questionnaire (i.e., PGSAS‐45) to evaluate postgastrectomy disorders, utilizing the Japanese version for clinical applications.^15^ According to the Japanese gastric cancer treatment guidelines, standard gastrectomy is defined as the resection of at least two‐thirds of the stomach, including D2 lymph node dissection.^11^ The PGSAS study showed that proximal gastrectomy promoted better QOL compared to total gastrectomy with Roux‐en‐Y reconstruction, leading to the widespread introduction of proximal gastrectomy. However, the optimal proximal gastrectomy reconstruction method has yet to be established.^10^, ^17^

Following the previous PGSAS study, “the PGSAS NEXT study,” a study on the optimal gastrectomy procedures to improve postoperative QOL for the cancer located at the upper third of the stomach or around the esophagogastric junction using the PGSAS‐45 questionnaire, was planned.

Various reconstruction methods have been utilized for proximal gastrectomy procedures. Japanese gastric cancer treatment guidelines have proposed three types of reconstruction methods for proximal gastrectomy: esophagogastrostomy, jejunal interposition, and double tract method.^11^ Analysis of the proximal gastrectomy cases collected in this study revealed that PGEG and PGDT were most popular reconstruction methods utilized in Japan.

Inada et al. reported that scores for esophageal reflux and dissatisfaction with meals were higher in patients who had not undergone an anti‐reflux procedure in the PGSAS study, which indicated that an appropriate anti‐reflux method is strongly recommended for PGEG.^18^ In the current study, 276 cases (93%) underwent anti‐reflex procedures for PGEG, whereas only 21 cases (7%) did not. The types of anti‐reflux methods for PGEG include the double flap method,^19^, ^20^ creation of pseudofornix and/or Hisoid angle, fundoplication,^21^, ^22^ and SOFY,^16^ all of which have been reported to have excellent anti‐reflux effects. The double flap method, which had been performed in 154 cases, had the best anti‐reflux effects for esophagogastric anastomosis with fundoplication based on valvuloplasty by creating the seromuscular double flaps, although this method is technically difficult and time consuming.^23^ PGDT requires 8–15 cm of interposed jejunum between the esophagus and the remnant stomach and an escape route using gastrojejunostomy.^24^, ^25^ The double tract method requires plural anastomosis (i.e., esophagojejunostomy, jejunogastrostomy, and jejunojejunostomy), although each anastomosis procedure is technically easy and familiar to many surgeons. Since the risk of an internal hernia is a concern, it is better to do suture closure of the mesentery gap caused by reconstruction.

From the background of both groups, the laparoscopic approach for proximal gastrectomy has been the mainstream approach for gastric cancer. In the previous PGSAS study, the laparoscopic surgery rate for proximal gastrectomy was only 17.2%,^10^ whereas that for proximal gastrectomy in the current PGSAS NEXT study, which was conducted nearly 9 years after the PGSAS study, has since increased. Currently in Japan, laparoscopic proximal gastrectomy is growing more popular than open proximal gastrectomy for early upper third gastric cancer. The JCOG1401 trial published in 2019 confirmed the safety of laparoscopic total/proximal gastrectomy for clinical stage I gastric cancer.^26^ An increasing number of facilities are introducing robotic surgery for stomach cancer.^27^ In particular, for upper gastric cancer and esophageal gastric junction cancer, the merit of performing robotic surgery is considered to be great because surgical operation can be performed easily and precisely in a narrow field of view, and its spread is expected.

Surgical options, such as the size of the remnant stomach, anastomosis method, adding procedures to create an anti‐reflux function, length of the resected abdominal esophagus, and preservation of the celiac branch of the vagus nerve, can influence the QOL after proximal gastrectomy. The PGDT group had a greater esophageal resection length and shorter distance from the diaphragm to the anastomosis site, indicating that PGDT had been employed for cases requiring longer esophagus resection lengths and allows anastomosis at a higher diaphragm level. The PGEG group had a significantly larger remnant stomach size and higher preservation rate of the celiac branch of the vagus nerve than the PGDT. Thus, the remnant stomach size affected the choice of the reconstruction method and the rate of function preserving procedure after proximal gastrectomy. Nonetheless, introducing an effective anti‐reflux method in cases with a small remnant stomach remains difficult; therefore, PGDT may be preferred in such cases.

In all cases, BW loss (%) tended to be smaller in the PGEG group than the PGDT group. The possible explanations for this include a larger remnant stomach size acting as a reservoir and the total passage of the ingested meal through the physiological duodenal route in the PGEG group. In contrast, the PGDT group had a rather small remnant stomach size, possibly inducing and postpancreaticocibal asynchrony if a significant proportion of the ingested meal directly passes through the escape jejunal route, which may result in nutrient indigestion. In PGDT, if food does not flow into the residual stomach, nutritionally it will be the same as total gastrectomy using Roux‐en‐Y reconstruction, so it is important to devise a jejunogastrostomy procedure so that more food flows into the residual stomach. Several devices have been reported, one of which is to increase length of jejunogastrostomy (6 cm or more). In the part of the jejunum used for jejunogastrostomy, the luminal structures necessary for peristalsis to transport food is lacking, so it is expected that food will flow into the residual stomach by lengthening this portion. In the report by Kamiya et al., the weight loss was less in the longer jejunogastrostomy group, namely, −12.5% in the longer group (6 cm or more), compared to −14.9% in the shorter group (5 cm or less). Although there was no significant difference.^28^

The current study showed that the QOL of the PGDT groups was slightly superior to that of the PGEG group across several main outcome measures of the PGSAS‐45 during stratified analysis according to remnant stomach sizes of around 1/2 and 2/3. Among those with a remnant stomach size of 1/2, the PGDT group was superior in three main outcome measures, namely constipation SS, dumping SS, and working ability, compared to the PGEG group. Similarly, among those with a remnant stomach size of 2/3, the PGDT group was superior in five main outcome measures, namely diarrhea SS, dissatisfaction with symptoms, dissatisfaction with daily life SS, constipation SS, and dissatisfaction with work. No difference in the other main outcome measures, including body weight loss, was observed between the groups.

The use of a fiberscope can be expected to improve the early diagnosis of upper third gastric cancer. The Japanese gastric cancer treatment guidelines recommended proximal gastrectomy for cN0, cT1 tumors where more than half of the distal stomach can be preserved.^11^ Though the present study showed that PGDT can promote slightly better QOL than PGEG, the reconstruction method for proximal gastrectomy can be selected according to each surgeon's institutional principles or discretion at this point. Given that the procedures employed for PGEG and PGDT are still incomplete, further techniques for improving both methods and studies revaluating PGEG and PGDT in the future are required. One limitation of the present study was its retrospective nature and the unbalanced number of patients in each group. A well‐designed prospective study should therefore be conducted in the future.

## 5 CONCLUSION

Our results indicated that the QOL of the PGDT group was slightly superior to that of the PGEG group for several main outcome measures of the PGSAS‐45 during stratified analysis according to remnant stomach sizes of around 1/2 and 2/3, with a small effect size. The revaluation of both reconstruction methods after compensating the shortcomings of each procedure (i.e., preventing esophageal reflux for PGEG and maintaining physiological food passage for PGDT) will be a substantial issue in the future.

## FUNDING INFORMATION

This study was supported by a grant from the Jikei University and the Japanese Gastric Cancer Association.

## CONFLICT OF INTEREST

Dr. H.M. is an editorial member at the Annals of Gastroenterological Surgery. The other authors declare that they have no conflicts of interest.

## ETHICS STATEMENTS

This study was approved by the local ethics committees of each participating institution and was in accordance with the ethical standards of the responsible committee on human experimentation and with the Helsinki Declaration of 1964 and later versions. Written informed consent was obtained from all enrolled patients. Trial registration: The University Hospital Medical Information Network Clinical Trials Registry (registration number 000032221).
